# Factors Associated with Delayed Menopause in Iran: Findings from Fasa Cohort Study, a Branch of Persian Cohort Study

**DOI:** 10.22086/gmj.v0i0.922

**Published:** 2018-02-23

**Authors:** Mojtaba Farjam, Zahra Amiri, Mehdi Sharafi, Ehsan Bahramali

**Affiliations:** ^1^Non-Communicable Diseases Research Center, Fasa University of Medical Sciences, Fasa, Iran; ^2^Shiraz University of Medical Sciences, Shiraz, Iran

**Keywords:** Delayed Menopause, Logistic Regression Analysis, Iran

## Abstract

**Background:**

The investigation of middle-aged women’s mental and physical health measures should be focused on menopause-a predictable physiological phenomenon in their lives. The prevalence of a majority of chronic diseases increases after this period. This study aimed to determine the risk factors of delayed menopause (climacterium tardum).

**Materials and Methods:**

The current cross-sectional research was conducted on 1930 menopausal women who were referred to the cohort study of Fasa University of Medical Sciences, Fars Province, Iran, during 2014-2015. The data were extracted from the database, and then the variables were checked for accuracy. Finally, the data were analyzed using logistic regression analysis.

**Results:**

The study population included 1930 menopausal women with the mean age of 57.98 ± 5.8 years. Of these, 1555 (80.6%) were married, and the rest were single and widowed. The mean age at menarche was 13.7 ± 1.64 years. Additionally, 1726 women (89.4%) had experienced natural menopause, whereas the rest had experienced delayed menopause. The results of the multivariate analysis indicated that delayed menopause was associated with marital status, education level, age at menarche, occupation, abortion, and use of contraceptive methods. However, no significant relationship was found between delayed menopause and smoking, duration of lactation, duration of using contraceptive pills, and number of childbirths.

**Conclusion:**

Considering the increased life expectancy among women, delayed menopause, and its risk factors should be taken into account. Although genetic factors play key roles in age at the onset of menopause, the role of sociodemographic factors such as marital status and pregnancy should not be ignored.

## Introduction

According to the World Health Organization (WHO), natural menopause is defined as the cessation of monthly menstruation in women, which occurs due to the loss of ovarian follicular activities and leads to the termination of fertility period in women. Therefore, a woman will be known as menopausal if she experiences 12 months of menstruation cessation-not attributable to pregnancy, lactation, or other hormonal disorders (1). Although menopause is a naturally occurring physiological phenomenon, with the global trend of population aging and the subsequent increase in the population of menopausal women, the health of women around the age of menopause is being considered an important health concern (2). The age of onset of menopause ranges from 45 to 55 years (3). The mean age of women experiencing menopause is 51 years, and just 4% of women experience menopause before the age of 40 years (4). In a study conducted in Iran in 2013, the mean age of menopause was 50.4 years; it was lower in rural than in urban areas. Additionally, the menopause age was higher in industrial societies than in nonindustrial and poor communities (5). Some researchers consider menopause age as a health index. Therefore, a better understanding of the factors affecting this age might provide some clinical and epidemiological insights. Also, women experiencing delayed menopause are exposed to the risk of breast and uterine cancers (6-8). Furthermore, menopause age is inversely related to the development of some noncommunicable diseases, including osteoporosis and cardiac disease. Therefore, this study aimed to identify the factors related to delayed menopause, with the hope that the results would improve the knowledge regarding the risk factors of this phenomenon. A better understanding of this issue can ultimately help promote the quality of life of middle-aged women as important members of the society, who mostly adopt outstanding responsibilities to take care of both younger and older populations.

## Materials and Methods

This cross-sectional study was conducted on 1930 menopausal women who were referred to Fasa University of Medical Sciences in Sheshdeh, Fasa, Fars Province, Iran, during 2014-2015. Fasa cohort study is conducted by Fasa University of Medical Sciences under the supervision of Persian National Cohort of the Research Deputy of the Ministry of Health, Treatment, and Medical Education, and it considers the factors affecting noncommunicable diseases in Iranian population (9-12). All the menopausal women referring to this center over a period of 1 year were enrolled into the study. Then, the relevant information was extracted from the database in this research center using a questionnaire whose validity and reliability were approved by a group of experts in the field. The required information was gathered via an oral interview. Thereafter, the data were entered into Microsoft Excel 2013, and the variables were checked for accuracy. If they did not match, then verifications were done by referring to the patients’ medical histories. After that, the data were analyzed using the SPSS statistical software, version 19. For the descriptive analysis of quantitative variables, we used mean and SD, and for the analytic comparisons, we used logistic regression (LR). First, the variables were entered into the model as univariate variables. Then, the variables with significance level less than 0.25 (P<0.25) were entered into the model as multivariate ones using the forward LR method to control the confounding variables. The variables included the menopause age, marital status, education level, smoking, using contraceptive methods, duration of using contraceptive pills, lactation and its duration, parity, and the number of live births and abortion. According to the Stages of Reproductive Aging Workshop, women with 12 months of amenorrhea and reporting menopause were defined as postmenopausal or otherwise as premenopausal (12).

The women participating in this study had reached the menopause age. On the basis of their menopause age, they were divided into two groups, namely, natural menopause age (women reaching menopause during the ages of 45-55 years) and delayed menopause age (women reaching menopause after the age of 55 years) (13).

## Results

This study was conducted on 1930 menopausal women with the mean age of 57.98 ± 5.8 years. Of these, 1555 (80.6%) were married, and the rest were single and widowed.

The mean age at menarche was 13.7 ± 1.64 years. Additionally, 1726 (89.4%) and 204 women (10.6%) had experienced natural and delayed menopause, respectively; 261 women (13.5%) were educated, and 371 ones (19.2%) had jobs.

Furthermore, 273 (14.1%) and 17 women (0.9%) used to smoke and used drugs, respectively. Considering socioeconomic status, the majority of the women (51.1%) belonged to economically average families.

According to the results, the median of live births was 7. Also, 449 women (24%) reported that they had experienced stillbirth. It should also be mentioned that 1326 participants had utilized contraceptive methods for an average of 34 ± 42.87 months. The mean duration of consuming oral contraceptive pills was also 33.26 ± 41.51 months.

Among the mothers, 1834 (95%) reported lactation for an average of 130.9 ± 61.06 months (Table-1). The results of crude and adjusted analyses of different variables with regard to delayed menopause are presented in Table-2. The results of the crude analysis revealed a significant relationship of delayed menopause with marital status, occupation, and use of contraceptives for more than three years. After adjustment, the probability of delayed menopause was 4.68 times higher in married women than others (odds ratio [OR] 4.68; 95% CI 1.87-11.69). Additionally, women who were homemakers were 2.73 times more exposed to delayed menopause than the working ones (OR 2.73; 95% CI 1.44-5.19). Furthermore, women whose menstruation started after the age of 15 years were 1.78 times more exposed to delayed menopause (OR 1.78; 95% CI 1.01-3.11). Regarding the relationship between delayed menopause and using contraceptive methods, the women who had used contraceptive methods for more than 3 years were 1.75 times more exposed to delayed menopause (OR 1.75; 95% CI 1.07-2.87). Moreover, the women who had experienced abortion were 1.84 times more exposed to delayed menopause (OR 1.84; 95% CI 1.24-2.78).

**Table-1:** Baseline Characteristics of the Menopausal Women

**Variables**	**No. (%) of participants**
**Age at Menopause (years)**	
<55	1726 (89.4)
>55	204 (10.6)
**Marital Status**	
Married	1555 (80.6)
Other	375 (19.4)
**Literacy**	
Illiterate	1621 (84)
Literate	309 (16)
**Occupation**	
Employee	371 (19.2)
Homemaker	1559 (80.8)
**Use of Contraceptive Methods**	
No	595 (31)
Yes	1326 (69)
**Child Birth**	
No	73 (3.8)
Yes	1857 (96.2)
**Lactation**	
No	96 (5)
Yes	1834 (95)
**Abortion**	
No	1424 (76)
Yes	449 (24)
**Stillbirth**	
No	1424 (76)
Yes	449 (24)
**Smoking**	
Never	1657 (85.9)
Current and former	273 (14.1)

**Table-2:** Crude and Adjusted Odds Ratio Estimates of Different Variables in Women with Delayed Menopause

**Variables**	**Crude**		**Adjusted**	
	OR (95% CI)	P-value	OR (95% CI)	P-value
**Marital status**				
Married	Reference			
Other	4.68 (2.52-8.70)	0.001	4.68 (1.87-11.69)	0.001
**Literacy**				
Illiterate	Reference			
Literate	1.36 (0.94-1.97)	0.09	1.71 (1.10-2.65)	0.01
**Occupation**				
Employee	Reference			
Homemaker	2.09 (1.32-3.31)	0.002	2.73 (1.44-5.19)	0.002
**Smoking**				
Never	Reference			
Current and former	1.007 (0.06-1.52)	0.97	*	*
**Use of Contraceptive Methods (years)**				
<3	Reference			
>3	1.77 (1.09-2.87)	0.02	1.75 (1.07-2.87)	0.02
**Use of Contraceptive Pills (years)**				
<5	Reference			
>5	0.79 (0.44-1.43)	0.44	*	*
**Child Birth**				
No	Reference			
Yes	1.33 (0.57-3.11)	0.50	*	*
**Abortion**				
No	Reference			
Yes	1.58 (1.15-2.18)	0.004	1.84 (1.24-2.78)	0.002
**Age at Menarche (years)**				
<15	Reference			
>15	1.35 (0.87-2.10)	0.17	1.78 (1.01-3.11)	0.04
**Duration of Lactation (years)**				
<4	Reference			
>4	1.63 (0.96-2.78)	0.06	*	*

*not included

A significant relationship was also found between the women’s education level and delayed menopause (OR 1.71; 95% CI 1.10-2.65).

## Discussion

This study aimed to identify the risk factors of delayed menopause in Fasa cohort study, regardless of its consequences. Generally, delayed menopause is crucial for both individuals and health systems. First, the hormonal and behavioral changes related to menopause lead to a high demand for health services.

Second, menopause introduces a major change in the morbidity pattern of women, especially in view of the presence of osteoporosis and cardiovascular problems, as well as an increased risk of gynecological cancers (14). Our study was conducted on 1930 menopausal women. Considering the relationship between marital status and delayed menopause, the results indicated that the risk of delayed menopause was 4.68 times higher in married women than others. This is consistent with the results of other studies conducted on the issue (15-17). However, Nagata *et al*. found no significant relationship between marital status and delayed menopause (18). According to Kamyabi *et al*., single women reach the menopause age much sooner than the married ones because of regular ovarian activities and incessant stimulation of follicles under the effect of pituitary hormones (19). The findings of the present study also demonstrated that the education level was significantly associated with delayed menopause, which is in agreement with the results of other studies (20,21) . The results of the research carried out by Ismaeeli *et al*. also showed that women’s education level was effective in early menopause (22). In contrast, Tavasoli *et al*. reported no significant relationship between the education level and menopause age (23). Some studies have revealed a statistically significant relationship between smoking and menopause age (24-26). However, some other studies, including the current one, did not yield similar results (27). According to Ayatollahi study, this finding can be attributed to the lack of such habits among Iranian women (28). Our study results indicated that women who are homemakers were 2.73 times more exposed to delayed menopause than those who worked outside, which is in contrast with the results obtained by Ortiz (29). However, the results of the research by Nohjah revealed no significant relationship between occupation and menopause age (30). The findings of the current study also suggest that women whose menarche age was over 15 years were exposed to a higher risk of delayed menopause.

Theoretically, earlier ovarian activities can be probably followed by an earlier menopause age. In other words, it seems that women with early onset of menarche will reach the menopause age much sooner (31,32). On the contrary, the results of the study by Koto *et al* showed no statistically significant relationship between menarche age and menopause age (33). In our study, although the percentage of delayed menopause was higher among women with a history of breastfeeding for over 4 years, the difference was not statistically significant. Similarly, Gurrido-Latorre *et al*. reported that the women with lactation duration of 2.1 years experienced delayed menopause (14). The present study results showed no statistically significant relationships between the menopause age and duration of using contraceptive pills, which is consistent with the findings of some previous studies (34). However, some other studies revealed a relationship between these 2 factors (35). Moreover, the women who had experienced abortion were 1.84 times more exposed to delayed menopause, which is not supported by the results of other studies (36). The strengths of this study included its high number of enrolled people from a region that could represent all people with a large sample size. However, the limitations of the study should not be overlooked. One of the limitations of the study was that the required information was gathered via individual reports and comments. Therefore, despite the attempts to gather precise information, the limitations associated with the use of individual-oriented reports cannot be ignored. Moreover, other effective factors affecting menopause age, such as family history, body mass index for the evaluation of obesity, estrogen levels, and thyroid function tests, were not taken into consideration in this study. Hence, further studies on these factors need to be conducted.

Overall, the paradoxical effects of delayed menopause regarding two important health issues-mortality and morbidity-should be taken into account to justify the risks of this phenomenon. By finding these risk factors, individualized medicine will work for every single patient. In other words, delayed menopause can be beneficial or hazardous to a patient carrying the risk factors of cardiovascular disorders or cancer. Therefore, for every woman with specific risk of cancer or cardiovascular diseases, menopause age can be an additional risk factor or a protective one. This issue awaits further clarification.

## Conclusion

The findings of this study disclosed that although genetic factors play significant roles with regard to menopause age, other social factors including marital status and pregnancy should not be neglected. Overall, the menopause age is of utmost importance in women’s lives, and some measures are needed to be introduced in order for health providers and women to enhance and clarify their viewpoints regarding this period. Also, essential training should be incorporated into health programs to help women spend their aging period more successfully in relatively healthy conditions.

## Conflict of Interest

The authors have no financial disclosures to declare and no conflicts of interest to report.
